# Loss of body weight is dose-dependently associated with reductions in symptoms of hip osteoarthritis

**DOI:** 10.1038/s41366-024-01653-w

**Published:** 2024-10-17

**Authors:** Zubeyir Salis, Ryan Gallagher, Luke Lawler, Amanda Sainsbury

**Affiliations:** 1 Division of Rheumatology, Geneva University Hospitals and Faculty of Medicine, University of Geneva, Geneva, Switzerland; 2 Prima Health Solutions, Sydney, New South Wales, Australia. A fully owned subsidiary of Honeysuckle Health, Newcastle, NSW Australia; 3 The University of Western Australia, School of Human Sciences, Perth, WA Australia

**Keywords:** Weight management, Epidemiology

## Abstract

**Background/objectives:**

While weight loss is recommended for managing hip osteoarthritis (OA), most evidence comes from knee OA studies, limiting its applicability to hip OA. This study addresses this gap by examining the effects of weight loss on hip OA symptoms.

**Design and setting:**

A retrospective audit of routinely collected healthcare data from participants enrolled in the Osteoarthritis Healthy Weight for Life (OAHWFL) program, designed for individuals with knee or hip OA.

**Participants:**

In total, 1714 adults with hip OA were selected from the OAHWFL program; 1408 completed the initial 18-week weight loss phase, while 306 did not complete it. After 18 weeks, participants transitioned to an indefinite weight maintenance phase.

**Exposure:**

Percentage change in body weight from baseline at 18 weeks.

**Outcomes:**

Changes in the five subscales of the Hip Disability and Osteoarthritis Outcome Score (HOOS) (Pain, Activity Limitations in Daily Living, Stiffness and Range of Motion, Sports and Recreation Function, and Hip-related Quality of Life) from baseline to 18 weeks.

**Statistics:**

Linear regression, adjusted for sex and baseline values of age, weight, and respective HOOS scores, assessed the relationship between percentage weight change (analyzed as both a continuous variable and in categories: ≤2.5%, >2.5–5.0%, >5.0–7.5%, >7.5–10%, and >10% of baseline weight) and changes in all five HOOS subscales.

**Results:**

At baseline, participants had a mean age of 65.14 years, 70% were female, and 78% were individuals with obesity (Body Mass Index ≥30 kg/m^2^). A statistically significant dose-response relationship was observed between weight loss and improvements in all HOOS subscales, with the greatest improvement in the Hip-related Quality of Life subscale (14.42 points, 31.14%) for >10% weight loss.

**Conclusion:**

Our findings suggest that weight loss is associated with reduced symptoms of hip OA, supporting weight loss as an effective treatment strategy for hip OA.

## Introduction

Osteoarthritis (OA) is a common and disabling condition, characterized by joint pain and stiffness due to gradual cartilage erosion [1]. Among its forms, hip OA is particularly debilitating, significantly limiting locomotor activity and functional ability, often progressing to a severity that necessitates joint replacement [2, 3]. In the past 30 years, the prevalence of hip OA has risen from 17.02 to 18.70 per 100,000 persons [4], a trend reflecting broader demographic changes such as an aging global population and increasing obesity rates [1]. Obesity is a well-documented risk factor for both the development and progression of hip OA, likely due to increased mechanical loading and systemic inflammation [5, 6]. The increasing prevalence of hip OA, exacerbated in populations with obesity, highlights the need for effective symptom management strategies to improve the quality of life for affected individuals [7].

Although weight loss is widely recommended for individuals with overweight and obesity in OA guidelines worldwide [8–13], the evidence supporting its efficacy specifically for hip OA remains limited, as the evidence is primarily drawn from studies on knee OA. As a result, there is a gap in the literature specifically addressing the impact of weight loss on hip OA [1, 7]. Previous research on the effects of weight loss on the symptoms of hip OA is limited and inconclusive [14–16]. A systematic review [14] analyzed nine observational studies assessing the impact of weight loss through bariatric surgery on joint pain in people with knee and hip OA. However, these studies did not exclusively target individuals with hip OA, leading to ambiguity regarding the specific effects on hip joint pain. While the review suggested a potential reduction in hip pain following bariatric surgery, definitive conclusions were hindered by limitations in the data. These included small sample sizes, an inconsistent focus on hip pain relative to knee pain, and methodological variability. Additionally, the high risk of bias in many of these studies further weakened the confidence in their results [14]. Similarly, a study [15] involving 35 participants with hip OA and overweight or obesity, examined the effects of exercise and weight loss over eight months. Although that study [15] found a 25.4% reduction in self-reported hip pain after an average weight loss of 5% at the end of the eight months, the small sample size (35 participants) precludes any conclusion about the potential efficacy of weight loss for pain in hip OA in this population. Another observational study, this one using the Osteoarthritis Initiative (OAI) data [16], did not find any association between change in body mass index (BMI) and the development or resolution of pain in hip OA over four years. However, it should be noted that the OAI cohort consists of people with or at risk of knee OA, and is not specifically targeted to people with hip OA. Given the limited and inconclusive findings from previous research, our study aims to explore the potential of weight loss as a treatment strategy for hip OA.

## Methods

### Study design and setting

This study is a retrospective audit of routinely collected healthcare data from participants enrolled in the Osteoarthritis Healthy Weight for Life Weight Loss (OAHWFL) program, designed for individuals diagnosed with knee or hip OA. The OAHWFL program, based in Australia, caters to both rural and urban residents, offering structured weight loss and weight management strategies and using mail replacement products such as shakes. The OAHWFL program is implemented by Prima Health Solutions Pty Ltd. The program runs on behalf of participating private health insurance providers. The full cost of the program, including that of meal replacement products, is covered by the private health insurance provider of the participant. We report the findings following the Consolidated Standards of Reporting Trial (CONSORT) guidelines [17] that are applicable to a non-randomized design.

### Study participants and enrollment procedure

A total of 1,714 adults who had a diagnosis of hip OA were selected from participants enrolled in the OAHWFL program between January 2014 and February 2023. Although the program includes individuals with both hip and knee OA, only those with hip OA were included in this study. There were several pathways for enrollment into the OAHWFL program. The majority of participants in this study were recruited via direct mail from their private health insurance provider. In this recruitment process, individuals identified by hospital claims data as having undergone a surgical hip procedure (such as arthroscopy or hip joint replacement) indicative of hip OA were mailed a detailed explanation of the OAHWFL program (with an invitation to join the program) by their private health insurance provider. The other pathways to recruitment were via advertisement in private health insurance provider channels (email, website, newsletter, magazine, coach/nurse), general practitioner or specialist referral, or word of mouth. Those who wanted to be enrolled in the program were required to obtain a written referral from their medical professional (either general practitioner/family physician, orthopedic surgeon, or rheumatologist) confirming their weight and height and diagnosis of hip OA.

### Ethical approval

Ethical approval was obtained from the University of Western Australia Research Ethics Committee (#2023/ET000355), and the requirement for written consent was waived.

### Intervention (the OAHWFL program)

The OAHWFL program was a remotely delivered intervention designed for individuals with knee or hip OA and consisted of two main phases: an initial 18-week weight loss phase followed by an indefinite long-term weight maintenance phase.

#### Weight loss phase (18 weeks)

The OAHWFL program commenced with an 18-week weight loss phase that included a portion control eating plan with staged use of a very low-calorie meal replacement product (KicStart VLCD®), and an activity plan. This phase was divided into three stages:

- Stage 1: Motivational Weight Loss (Weeks 1–6): Participants replaced two meals per day (usually breakfast and lunch) with KicStart VLCD® meal replacement products while consuming “free foods” such as berries and leafy greens. The third meal (typically dinner) was portion-controlled.
- Stage 2: Consolidation Weight Loss (Weeks 7–12): Participants replaced one meal per day (usually breakfast) with a KicStart VLCD® meal replacement, while the remaining two meals (usually lunch and dinner) were portion-controlled. “Free foods” were still allowed, as in Stage 1.
- Stage 3: Short-Term Weight Maintenance (Weeks 13–18): Participants transitioned to eating portion-controlled meals for breakfast, lunch, and dinner without meal replacements, supplemented by “free foods”.

During the first six weeks, participants were also provided with an in-home strength, mobility, and pain management kit, designed for individuals with knee and/or hip pain and reduced mobility. The kit supports participants in establishing a home-based routine for muscle strengthening, mobility, and education, whether they are managing their condition independently or under the care of a physiotherapist.

#### Long-term maintenance phase (indefinite)

After completing the initial 18-week weight loss phase, participants transitioned to an indefinite weight maintenance phase. In this phase, they were encouraged to continue with portion-controlled, whole-food-based meals supplemented with “free foods” and to integrate the principles learned during the program into their daily lives.

Throughout both phases, participants received additional educational resources, motivation, support, and advice via online platforms or by phone, provided at predetermined intervals and upon request.

While a minimum of 5% weight loss is generally recommended for symptom relief in OA [9] (this recommendation is based on studies targeting knee OA), the OAHWFL program aimed for a more substantial 7–10% reduction in body weight over its 18-week weight loss phase. Previous research [18] has demonstrated the effectiveness of the OAHWFL program in achieving such significant weight loss for individuals with knee OA. In that study [18], 1383 participants were enrolled, and on average, they experienced an 8.3% reduction in their baseline body weight at the end of the 18-week weight loss phase. Of the total cohort of 1383 participants, 1,304 (94%) achieved a > 2.5% reduction in body weight, and 431 (31%) had a > 10% reduction over the 18-week weight loss phase [18].

Our study focuses on the outcomes of this initial 18-week weight loss phase, although participants continued to receive support for maintaining reduced weight beyond this period.

### Exposure

The primary exposure in our study was the percentage change in body weight from baseline at 18 weeks after commencement of the OAHWFL program (i.e., at the end of the weight loss phase). Participants self-reported their body weight at both time points. Additionally, their general practitioners verified baseline body weight. We opted to use the percent change in body weight as the exposure instead of the change in BMI because height remains constant over the 18-week weight loss phase, and as a result, it makes no difference to the results (and conclusions) whether the percentage body weight change or BMI is used in the analyses, but using percent body weight change simplifies interpretation.

### Outcomes

Our primary outcomes were changes in scores of the five subscales of the Hip Disability and Osteoarthritis Outcome Score (HOOS) from baseline to the end of the 18-week weight loss phase [19]. The HOOS is a validated tool, and it has been noted for its reliability and sensitivity to changes in conditions of hip OA [19]. The questionnaire and a user guide are accessible at http://www.koos.nu. The HOOS encompasses 40 items distributed across the five subscales, all of which are relevant to patients: Pain (10 items); Activity Limitations in Daily Living (17 items); Stiffness and Range of Motion (5 items); Sports and Recreation Function (4 items); and Hip-related Quality of Life (4 items). Responses are captured on a 5-point Likert scale ranging from 0 (no issues) to 4 (extreme issues). The overall scores for each of the five subscales were calculated by summing the responses, and these were then linearly transformed to a scale ranging from 0 (worst possible condition) to 100 (best possible condition).

As a secondary outcome, we assessed the minimal clinically important improvement (MCII) in the functional score of the Western Ontario and McMaster Universities Osteoarthritis Index (WOMAC) [20]. MCII was defined as a change of 7.9 units on a 0–100 scale of the functional score of WOMAC [21]. We utilized HOOS data to derive WOMAC scores, as the HOOS contains all WOMAC questions in their unchanged form [20]. Specifically, the subscale of ‘Activity Limitations in Daily Living’ of the HOOS corresponds to the functional score in WOMAC [19]. Higher scores on the WOMAC indicate more severe functional disabilities associated with hip OA, contrasting with the HOOS scoring convention outlined above.

### Statistical analyses

Of the 1714 participants diagnosed with hip OA initially selected from the OAHWFL program, 1408 completed the 18-week weight loss phase and provided complete data, while 306 did not. Our primary analysis included only the 1408 participants who completed the 18-week weight loss phase and provided complete data. We examined the dose-response relationship between percentage body weight change and each of the five subscales of the HOOS by conducting linear regression analyses, using percentage body weight change as a continuous variable from baseline to the end of the 18-week weight loss phase. The models were adjusted for sex, and the baseline measures of age, weight, and respective HOOS scores.

In addition to using body weight change as a continuous variable, we analyzed the dose-response relationship between percentage weight change and changes in each of the five subscales of the HOOS using categories of percentage body weight change (categorized as ≤2.5%, >2.5–5.0%, >5.0–7.5%, >7.5–10%, and >10% body weight loss). This categorization is based on a precedent study [18] that examined body weight loss and symptoms of knee OA, but our participant cohort was distinct from that of the precedent study [18]. The ≤2.5% category of percentage body weight loss includes participants who experienced either a weight gain or no change in body weight. To assess for any dose-response relationship between categories of percentage body weight change and changes in HOOS subscales, we applied polynomial contrasts to the ordered categories of percentage body weight change within a linear regression framework. The model controlled for sex, and the baseline measures of age, weight, and respective HOOS scores, as in the analysis that used percentage body weight change as a continuous variable. We further calculated and reported the average mean change in the total HOOS score—representing the sum of all five HOOS subscale scores—across each category of percentage body weight change. Additionally, we reported the number and percentage of participants in each category of percentage body weight change who achieved the MCII in the WOMAC function score. To evaluate for any potential dose-response relationship between categories of percentage body weight change and percentages of participants achieving MCII, we used univariable polynomial contrasts within a linear regression framework. We have also repeated the analyses using categories of percentage body weight change for the participants who did not complete the 18-week weight loss phase. Moreover, we conducted a subgroup analysis on participants aged 65 years or older to evaluate whether body weight change impacts this population across all measured outcomes, considering the increased risk of frailty, the potential for different physiological responses, and a higher prevalence of comorbidities in older adults. We used R (version 4.3.1) for our analyses. We set our threshold for statistical significance as a two-tailed *P* value of less than 0.05.

### Sensitivity analysis

In our primary analysis, we included only participants who provided data at both baseline and the end of the 18-week weight loss phase. To evaluate the impact of missing data, we conducted a sensitivity analysis that included all participants, regardless of whether they completed the 18-week weight loss phase. While the HOOS scores were missing for those who did not complete the 18-week weight loss phase, we still had the final weight measurements for all participants, regardless of their completion status. This was possible because some participants submitted their final weight data via hardcopy forms after being marked as discontinued during the 18-week weight loss phase. These submissions were recorded in their profiles without changing their status to complete. For this sensitivity analysis, we used multiple imputations with chained equations to estimate the missing HOOS scores for participants who did not complete the phase. We used the Multivariate Imputation by Chained Equations (mice) package in R for multiple imputations with chained equations (20 samples, predictive mean matching algorithm). We then compared the imputed results with those from our primary analysis to assess the robustness of our findings.

## Results

### Baseline characteristics of individuals who participated in the OAHWFL program

Table 1 shows the baseline characteristics of participants who completed the 18-week weight loss phase as well as that of participants who did not complete it, as defined by whether they provided data at baseline and at the end of the 18-week weight loss phase. Of the 1714 individuals who were initially approved by their medical doctor to participate in the OAHWFL program, 1408 participants (82.15%) completed the 18-week weight loss phase, and 306 (17.85%) did not (Table 1).

**Table 1:** Demographic and clinical characteristics of the study participants at baseline. Values are the mean ± SD unless indicated otherwise.

Baseline characteristics	Participants who completed the 18-week weight loss phase of the OAHWFL program (*n* = 1408)	Participants who did not complete the 18-week weight loss phase of the OAHWFL program (*n* = 306)
Age, years	65.14 ± 9.42 (range: 30–90) Missing: none	59.81 ± 9.94 (range: 30–81) Missing: none
Gender, female participants, no. (%)	985 (69.96) Missing: none	231 (75.50) Missing: none
Weight, kg	94.66 ± 17.26 (range: 58.00–190.50) Missing: none	98.11 ± 19.67 (range: 63.70–182.50) Missing: none
Height, meters	1.66 ± 0.09 (range: 1.43–1.95) Missing: none	1.67 ± 0.09 (range: 1.42–2.00) Missing: none
BMI, kg/m^2^	34.12 ± 5.19 (range: 25.91–58.43) Missing: none	35.17 ± 5.83 (range: 25.71–57.13) Missing: none
Obesity (BMI ≥ 30 kg/m^2^) at baseline, no. (%)	1098 (77.98)	249 (81.37)
HOOS Pain subscale	61.98 ± 18.35 (range: 6–100) Missing: none	56.43 ± 19.22 (range: 14–100) Missing: none
HOOS Activity Limitations in Daily Living subscale	63.19 ± 18.77 (range: 4–100) Missing: none	59.07 ± 20.25 (range: 12–100) Missing: none
HOOS Stiffness and Range of Motion subscale	65.62 ± 18.45 (range: 0 –100) Missing: 5 (0.35%)	60.67 ± 20.09 (range: 5–100) Missing: none
HOOS Sports and Recreation Function subscale	44.54 ± 25.66 (range: 0–100) Missing: 18 (1.28%)	42.00 ± 27.24 (range: 0–100) Missing: none
HOOS Hip-related Quality of Life subscale	45.82 ± 21.18 (range: 0–100) Missing: 13 (0.92%)	41.20 ± 23.10 (range: 0–100) Missing: none
Total HOOS Score	280.68 ± 88.99 (range: 33–500) Missing: 24 (1.70%)	259.36 ± 96.87 (range: 64–500) Missing: none

*BMI* Body Mass Index, *HOOS* Hip Injury and Osteoarthritis Outcome Score, *OAHWFL* Osteoarthritis Healthy Weight for Life weight loss program, *SD* standard deviation.

At baseline, participants who completed the 18-week weight loss phase had a mean ± SD age of 65.14 ± 9.42 years. At baseline, the majority of participants who completed the 18-week weight loss phase were female (*n* = 985, 69.96%), and were individuals with a BMI in the obesity category (*n* = 1098, 77.98%, mean ± SD BMI of 34.12 ± 5.19 kg/m^2^) (Table 1). Participants who completed the 18-week weight loss phase reported moderate baseline scores for three of the five subscales in the HOOS, namely Pain, Activity Limitations in Daily Living, and Stiffness and Range of Motion. In contrast, scores were considerably lower (worse) for the other two of the five subscales, namely Sports and Recreation Function, and Hip-related Quality of Life (Table 1).

Compared to the group of participants who completed the 18-week weight loss phase, the group of participants who did not complete it, on average, was younger (59.81 ± 9.94 years), had a higher percentage of females (75.50%), and was primarily composed of individuals with a higher percentage with obesity (81.37%, with mean ± SD of BMI being 35.17 ± 5.83 kg/m^2^) at baseline (Table 1). The group of participants who did not complete the 18-week weight loss phase had consistently lower baseline scores across all five HOOS subscales compared to the group of participants who completed this phase. This suggests that participants who did not complete the 18-week weight loss phase began with more severe challenges from their hip OA (Table 1).

### Weight loss during the 18-week weight loss phase of the OAHWFL program

Among the participants who completed the 18-week weight loss phase, 1,291 (92%) achieved a weight loss of >2.5% of their initial body weight by the end of the 18 weeks (Table S1). Notably, approximately one-quarter of the participants (*n* = 356, 25%) achieved a weight loss of >10%. The mean ± SD weight loss of the participants who completed the 18-week weight loss phase was 7.19 ± 4.26 kg, representing a 7.55 ± 3.99% loss from baseline body weight (Table S1).

Among the participants who did not complete the 18-week weight loss phase, 147 (48%) achieved a body weight loss of >2.5% of their initial body weight during that period (Table S1). Only a small number of participants (*n* = 10, 3% and *n* = 7, 2%) achieved a body weight loss of >7.5–10% and >10%, respectively. The mean ± SD body weight loss of the participants who did not complete the 18-week weight loss phase was 2.63 ± 3.78 kg, representing a 2.67 ± 3.69% loss from baseline body weight (Table S1).

### Relationship between percentage body weight loss and changes in HOOS subscales during the 18-week weight loss phase

The dose-response relationships between percentage body weight change and improvements in all HOOS subscales were observed when analyzing percentage body weight change as a continuous variable in regression models (Table 2). The estimates showed that a 1% body weight loss from baseline would yield a 0.54 unit reduction (improvement) (95% CI: 0.36–0.72 units) in the HOOS Pain subscale; a 0.56 unit reduction (95% CI: 0.38–0.74) in the HOOS Activity Limitations in Daily Living subscale; a 0.43 unit reduction (95% CI: 0.25–0.62) in the HOOS Stiffness and Range of Motion subscale; a 0.77 unit reduction (95% CI: 0.48 to 1.06) in the HOOS Sports and Recreation Function subscale, and a 0.67 unit reduction (95% CI: 0.45–0.89) in the HOOS Hip-related Quality of Life subscale (Table 2).

**Table 2:** Relationship between percentage body weight change as a continuous variable and changes in HOOS scores.

Outcome	Weight loss (%) β (95% CI)	*P* value*
Change in HOOS Pain subscale after weight loss	0.54 (0.36–0.72)	**<0.001**
[% change from baseline]	[0.96 (0.56–1.36)]	**[<0.001]**
Change in HOOS Activity Limitations in Daily Living subscale after weight loss.	0.56 (0.38–0.74)	**<0.001**
[% change from baseline]	[0.91 (0.47–1.34)]	**[<0.001]**
Change in HOOS Stiffness and Range of Motion subscale after weight loss	0.43 (0.25–0.62)	**<0.001**
[% change from baseline]	[1.02 (0.59–1.45)]	**[<0.001]**
Change in HOOS Sports and Recreation Function subscale after weight loss	0.77 (0.48–1.06)	**<0.001**
[% change from baseline]	[2.37 (0.88–3.85)]	**[0.002]**
Change in HOOS Hip-related Quality of Life subscale after weight loss	0.67 (0.45–0.89)	**<0.001**
[% change from baseline]	[1.85 (0.66–3.04)]	**[0.002]**
Change in Total HOOS score after weight loss	2.98 (2.12–3.84)	**<0.001**
[% change from baseline]	[1.28 (0.88–1.67)]	**[<0.001]**

*Adjusted for sex, and the baseline measures of age, weight, and respective Hip Disability and Osteoarthritis Outcome Score (HOOS) scores. The β coefficients were calculated and reported based on 1% weight loss from baseline at the 18-week weight loss phase.

*CI* confidence intervals.

**Fig. 1:**
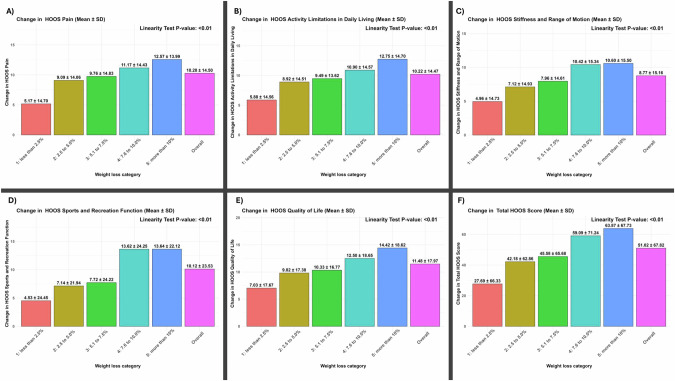
Dose-response relationship between the categories of percentage body weight change and changes in HOOS scores. Linearity tests were adjusted for sex and baseline values of age, weight, and respective HOOS subscale scores. *P* values less than 0.05 signify a linear dose-response relationship. A positive difference for change in Hip Disability and Osteoarthritis Outcome Score (HOOS) subscales indicates an improvement. SD standard deviation.

Similar to the results observed when percentage body weight change was treated as a continuous variable, a statistically significant dose-response relationship was also found between percentage body weight change when treated as a categorical variable and changes across all five HOOS subscales (Fig. 1A–E). The largest changes (improvements) in HOOS subscale scores were recorded in participants in the >10% body weight loss category, ranging from 10.60 ± 15.50 to 14.42 ± 18.62 points from baseline. Regarding the HOOS total scores, we also noted a significant dose-response relationship between the extent of body weight loss and the mean change in score. The greatest mean change (improvement) was 63.87 ± 67.73, observed in participants in the >10% body weight loss category (Fig. 1F).

**Fig. 2:**
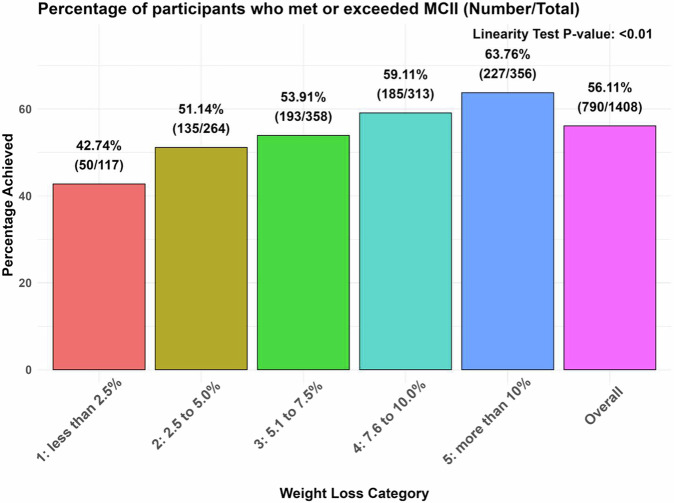
Dose-response relationship between the categories of percentage body weight change and the percentage of participants meeting or exceeding the MCII threshold for WOMAC function. The linearity test was not adjusted. A *P* value less than 0.05 signifies a linear dose-response relationship. MCII Minimal clinically important improvement, WOMAC Western Ontario and McMaster Universities Osteoarthritis Index.

Of the participants who completed the 18-week weight loss phase (*n* = 1408), 790 (56.11%) achieved or surpassed the MCII threshold for WOMAC function score, indicating that more than half of the participants experienced clinically meaningful improvements in functional abilities in their daily lives (Fig. 2). A statistically significant dose-response relationship was evident between the percentage of participants meeting or exceeding the MCII threshold for WOMAC function and the percentage of body weight change. Notably, the highest proportion of individuals achieving the MCII threshold (227 individuals, 63.76%) was found in those participants in the category of the highest body weight loss (i.e., >10% body weight loss) (Fig. 2).

The subgroup analyses on participants aged 65 years or older yielded results similar to those of our primary analyses in demonstrating a statistically significant dose-response relationship between the percentage body weight change and all HOOS subscales (Figs. S1A–S1.F and Fig. S2).

Among those participants who did not complete the 18-week weight loss phase, we observed a statistically significant dose-response relationship between the percentage body weight change and the HOOS subscales of Activity Limitations in Daily Living (Fig. S3.B), Sports and Recreation Function (Fig. S3.D), Hip-related Quality of Life (Fig. S3.E), as well as the Total HOOS score (Fig. S3.F), but not in the HOOS subscale of Stiffness and Range of Motion (Fig. S3.C). For the HOOS Subscale of Pain, a borderline statistically significant dose-response relationship was observed (Fig. S3.A). However, no dose-response relationship was observed for the individuals achieving the MCII threshold (Fig. S4). In the category of participants who lost more than 10% of their initial body weight, seven participants had data for the HOOS Pain subscale, but only one participant had data for the other HOOS subscales, which limits our ability to draw firm conclusions about these relationships from the participants who did not complete the weight loss phase.

### Sensitivity analyses

The results from sensitivity analyses performing multiple imputations using percentage body weight change as a continuous variable (Table S2) and body weight change as a categorical variable (Figs. S5.A–S1.F, and Fig. S6) were the same or similar to our primary analysis.

## Discussion

Our study demonstrated a clear correlation between weight loss and marked improvements in a range of symptoms associated with hip OA including pain, activity limitations in daily living, stiffness and range of motion, sports and recreation function, and hip-related quality of life. We observed a dose-response relationship, indicating that greater weight loss led to more substantial improvements, such that individuals who lost more than 10% of their body weight had greater improvements than those who lost less. These improvements were not only statistically significant but also translated into clinically meaningful enhancements in WOMAC function scores. Thus, our findings suggest that weight loss could reduce the symptoms of hip OA, supporting the potential of weight loss as an effective treatment strategy for hip OA.

While our study did not directly investigate the mechanisms through which body weight loss alleviates hip OA symptoms, several pathways could be considered, including reduced mechanical stress on the hip joint and decreased systemic inflammation associated with obesity [22]. Moreover, the potential for increased physical activity following body weight reduction [23] could also contribute to the observed improvements in hip OA symptoms, creating a positive feedback loop that reinforces the benefits of weight loss. Literature suggests that individuals with OA often engage in more physical activities following body weight loss due to reduced pain, improved mobility, and reduced fear of movement [24].

Our findings emphasize that significant weight loss is achievable in community settings, which can lead to meaningful improvements in symptoms for individuals with hip OA. Community-based programs are particularly vital as they offer accessible, adaptable interventions that can cater to the needs of the local population. Moreover, our study highlights the potential for remotely delivered interventions, such as digital health platforms and telehealth services, to support weight loss.

The dose-response relationship revealed by our study suggests that even modest body weight reduction can be beneficial for hip OA symptoms, with greater losses yielding more significant improvements. Therefore, public health policies should advocate for weight loss support systems that help adults with overweight or obesity and who also have hip OA. These interventions must be sustainable, supporting behavioral changes in the long term, and integrating into routine care through healthcare policies, thus making effective weight management a core component of treatment for patients with overweight or obesity and who also have hip OA. While our study demonstrates an associative beneficial effect of weight loss for hip OA even among participants aged 65 years or older, it should be noted that weight loss is associated with increased health risks in older adults [25], such as an increased risk of hip fracture [26, 27], increased risk of mortality [28], and higher risk of functional impairment and incident disability [29]. Therefore, caution is required when weight loss is advised for older adults.

This study presents several limitations that merit attention. First, this is an observational study of adults who were losing weight in a weight-loss program and not a randomized controlled trial, therefore, the results are associative not causative. Second, the weight-loss program included multifaceted approaches such as dietary components, exercise routines including strength training, personal support, and pain management strategies. We were unable to isolate the effects of these individual components on the outcomes, such as the effect of an increase in strength on the outcomes, raising the possibility that they may have contributed to the improvements observed across. A third limitation is the reliance on self-reported body weight measurements for the 18-week time point. Despite previous research [30] suggesting a high correlation between self-reported and actual measured body weight, the potential for error cannot be entirely discounted. Similarly, our study exclusively utilized patient-reported outcome measures to assess changes in symptoms and functional limitations related to hip OA severity, as data for other objective indicators, such as imaging or the need for total hip arthroplasty (THA), were unavailable. Future studies could benefit from including these additional measures to provide a more comprehensive evaluation of hip OA severity. Fourth, the long-term effects of the weight loss remain undetermined since data beyond the 18-week weight loss phase were not accessible, precluding any commentary on the sustainability of the weight loss achieved or its effects on the symptoms of hip OA. Lastly, the lack of socioeconomic data on the participants also constitutes a limitation. Although this does not detract from our findings, it limits our understanding of whether the weight loss results observed can be generalized across different socioeconomic strata. This information could be important in assessing the broader applicability and reach of the weight-loss program in diverse populations.

In conclusion, our study provides evidence that weight loss is associated with reduced symptoms of hip OA and, therefore, could be therapeutically beneficial for adults with overweight or obesity and who also have hip OA. While even small reductions in weight offer benefits, greater weight loss should be encouraged for substantial improvements in hip OA symptoms. Future randomized controlled trials comparing weight loss outcomes between intervention and control groups are needed to isolate the specific impact of weight loss on hip OA symptoms and further substantiate these findings.

## Supplementary information

Supplementary Materials

## Data Availability

The datasets generated and analyzed during the current study are not publicly available due to them being under the ownership of Honeysuckle Health. Aggregate data may be made available to researchers for scientific use upon written request. Requests must clearly articulate the scientific purpose and intended use of the data. Approval for data access is at the discretion of Honeysuckle Health (https://www.honeysucklehealth.com.au) and is subject to confidentiality agreements and other conditions deemed necessary by the organization.
