# Supplementary material for: Loss of body weight is dose-dependently associated with reductions in symptoms of hip osteoarthritis
Source: Int J Obes (Lond). 2024 Oct 17;49(1):147–53. doi: 10.1038/s41366-024-01653-w (PMC11683002; doi:10.1038/s41366-024-01653-w)
Supplement: Supplementary file 1 — Supplementary Materials [file 41366_2024_1653_MOESM1_ESM.docx]

**Loss of Body Weight is Dose-Dependently Associated with Reductions in Symptoms of Hip Osteoarthritis**

Zubeyir Salis*^1^, Ryan Gallagher ^2^, Luke Lawler^2^, Amanda Sainsbury^3^

^1^. Division of Rheumatology, Geneva University Hospitals and Faculty of Medicine, University of Geneva, Geneva, Switzerland

^2^. Prima Health Solutions, Sydney, New South Wales, Australia. A fully owned subsidiary of Honeysuckle Health, Newcastle, New South Wales, Australia.

^3^. The University of Western Australia, School of Human Sciences, Perth, WA, Australia

**Supplementary Materials**

Contents

[Figure S1. Subgroup analyses for participants 65 years or older for the dose-response relationship between the categories of percentage body weight change and changes in HOOS scores. 2](#_Toc177030089)

[Figure S2. Subgroup analysis for participants 65 years or older for the dose-response relationship between the categories of percentage body weight change and the percentage of participants meeting or exceeding the MCII threshold for WOMAC function 4](#_Toc177030090)

[Figure S3. Dose-response relationship between the categories of percentage body weight change and changes in HOOS scores for the participants who did not complete the 18-week weight loss phase 6](#_Toc177030091)

[Figure S4. Dose-response relationship between the categories of percentage body weight change and the percentage of participants meeting or exceeding the MCII threshold for WOMAC function for the participants who did not complete the 18-week weight loss phase of the OAHWFL program 8](#_Toc177030092)

[Figure S5. Sensitivity analysis. Dose-response relationship between the categories of percentage body weight change and changes in HOOS scores using multiple imputations for missing values 10](#_Toc177030093)

[Figure S6. Sensitivity analysis. Dose-response relationship between the categories of percentage body weight change and the percentage of participants meeting or exceeding the MCII threshold for WOMAC function using multiple imputations for missing values 12](#_Toc177030094)

[Table S1. Weight loss during the 18-week weight loss phase 14](#_Toc177030095)

[Table S2. Sensitivity analysis. Relationship between percentage body weight change as a continuous variable and changes in HOOS scores using multiple imputations for missing values. 15](#_Toc177030096)

Figure S1. Subgroup analyses for participants 65 years or older for the dose-response relationship between the categories of percentage body weight change and changes in HOOS scores. Linearity tests were adjusted for sex and baseline values of age, weight, and respective HOOS subscale scores. P-values less than 0.05 signify a linear dose-response relationship. A positive difference for change in Hip Disability and Osteoarthritis Outcome Score (HOOS) subscales indicates an improvement. SD: Standard Deviation.


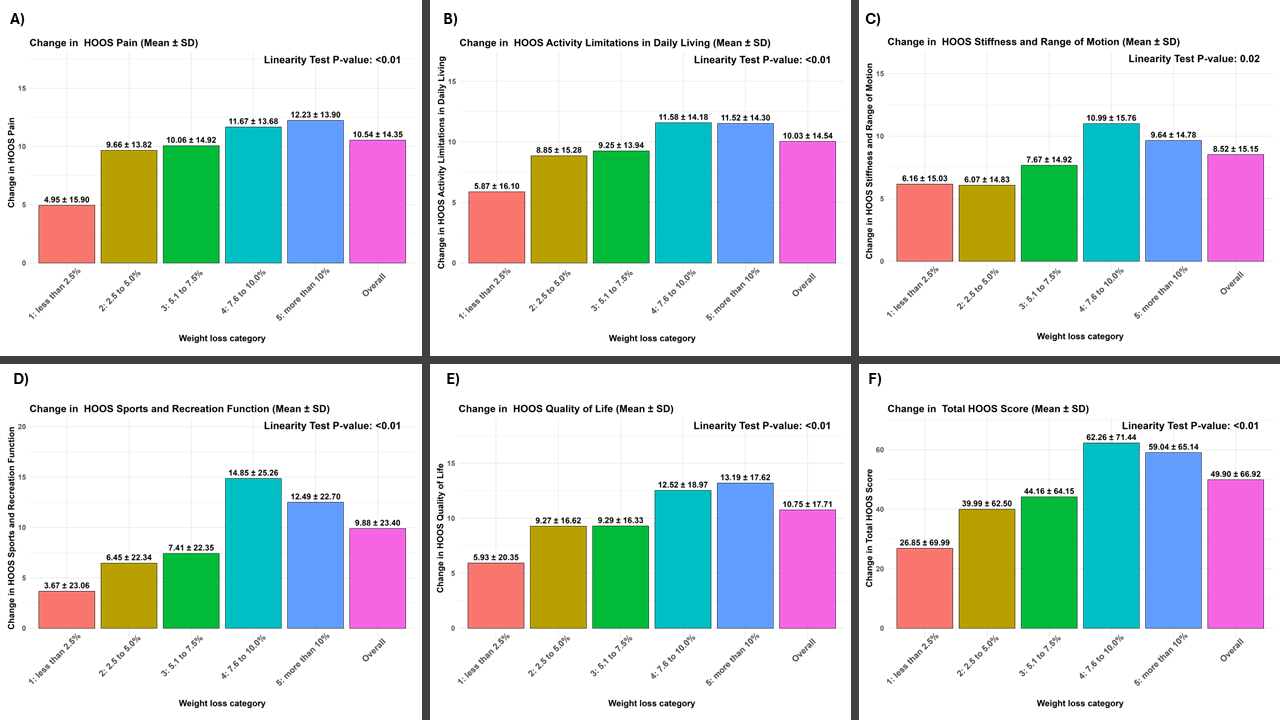


Figure S2. Subgroup analysis for participants 65 years or older for the dose-response relationship between the categories of percentage body weight change and the percentage of participants meeting or exceeding the MCII threshold for WOMAC function. The linearity test was not adjusted. A P-value less than 0.05 signifies a linear dose-response relationship. MCII: Minimal clinically important improvement; WOMAC: Western Ontario and McMaster Universities Osteoarthritis Index.


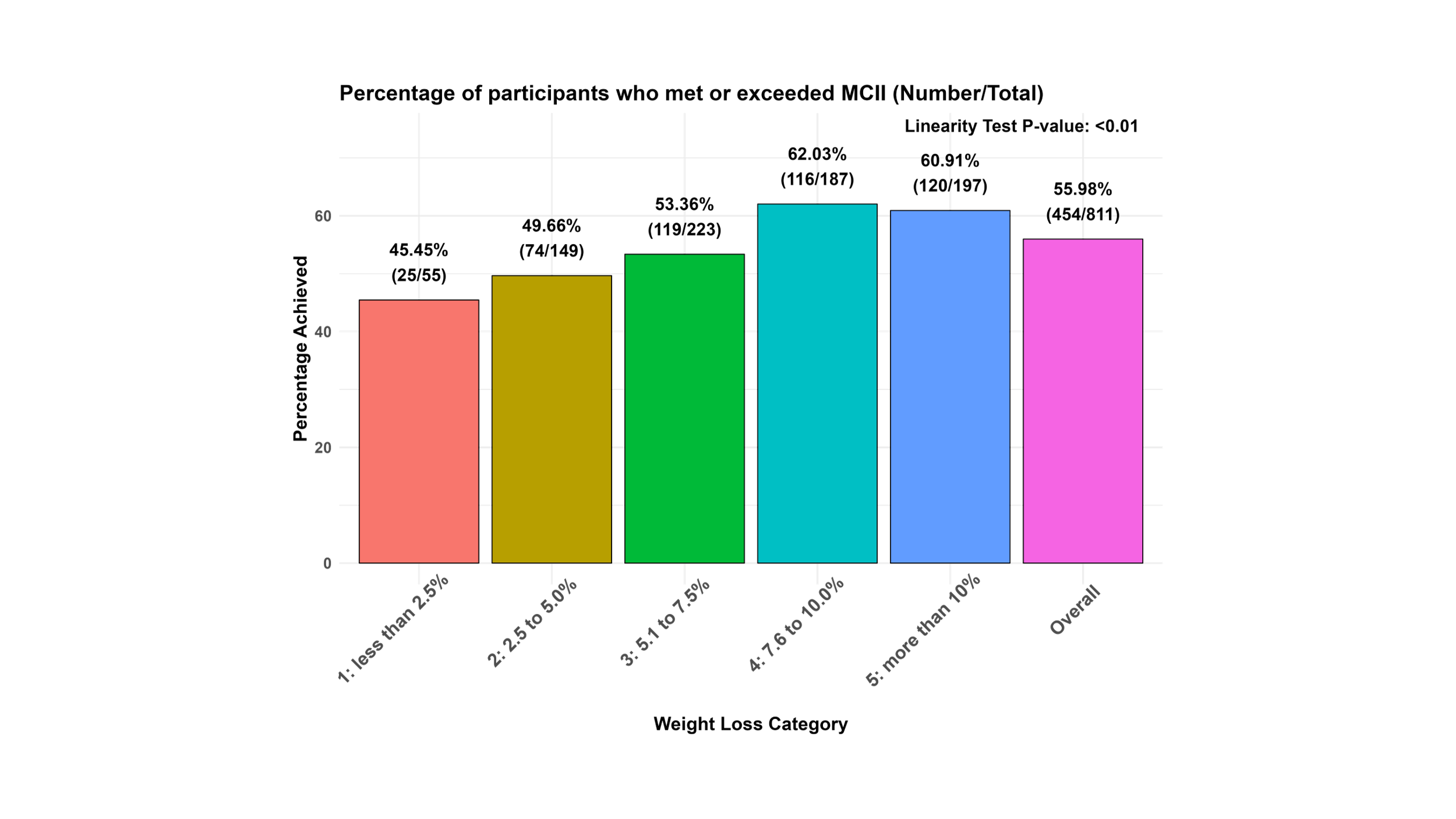


Figure S3. Dose-response relationship between the categories of percentage body weight change and changes in HOOS scores for the participants who did not complete the 18-week weight loss phase. Linearity tests were adjusted for sex and baseline values of age, weight, and respective HOOS subscale scores. P-values less than 0.05 signify a linear dose-response relationship. The SD for the category of more than 10% weight change in the HOOS subscales, excluding the HOOS Pain subscale, is denoted as NA due to only one participant. A positive difference for change in Hip Disability and Osteoarthritis Outcome Score (HOOS) subscales indicates an improvement. SD: Standard Deviation.


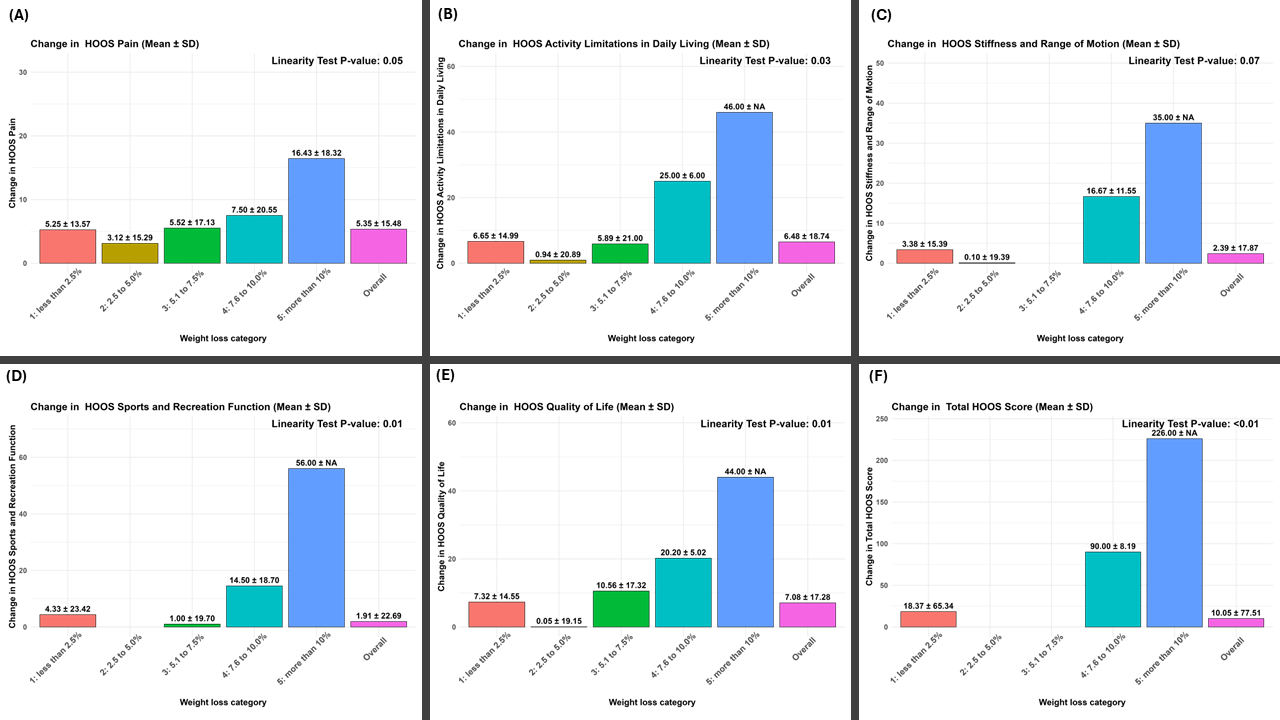


Figure S4. Dose-response relationship between the categories of percentage body weight change and the percentage of participants meeting or exceeding the MCII threshold for WOMAC function for the participants who did not complete the 18-week weight loss phase of the OAHWFL program. The linearity test was not adjusted. A P-value less than 0.05 signifies a linear dose-response relationship. MCII: Minimal clinically important improvement; WOMAC: Western Ontario and McMaster Universities Osteoarthritis Index.


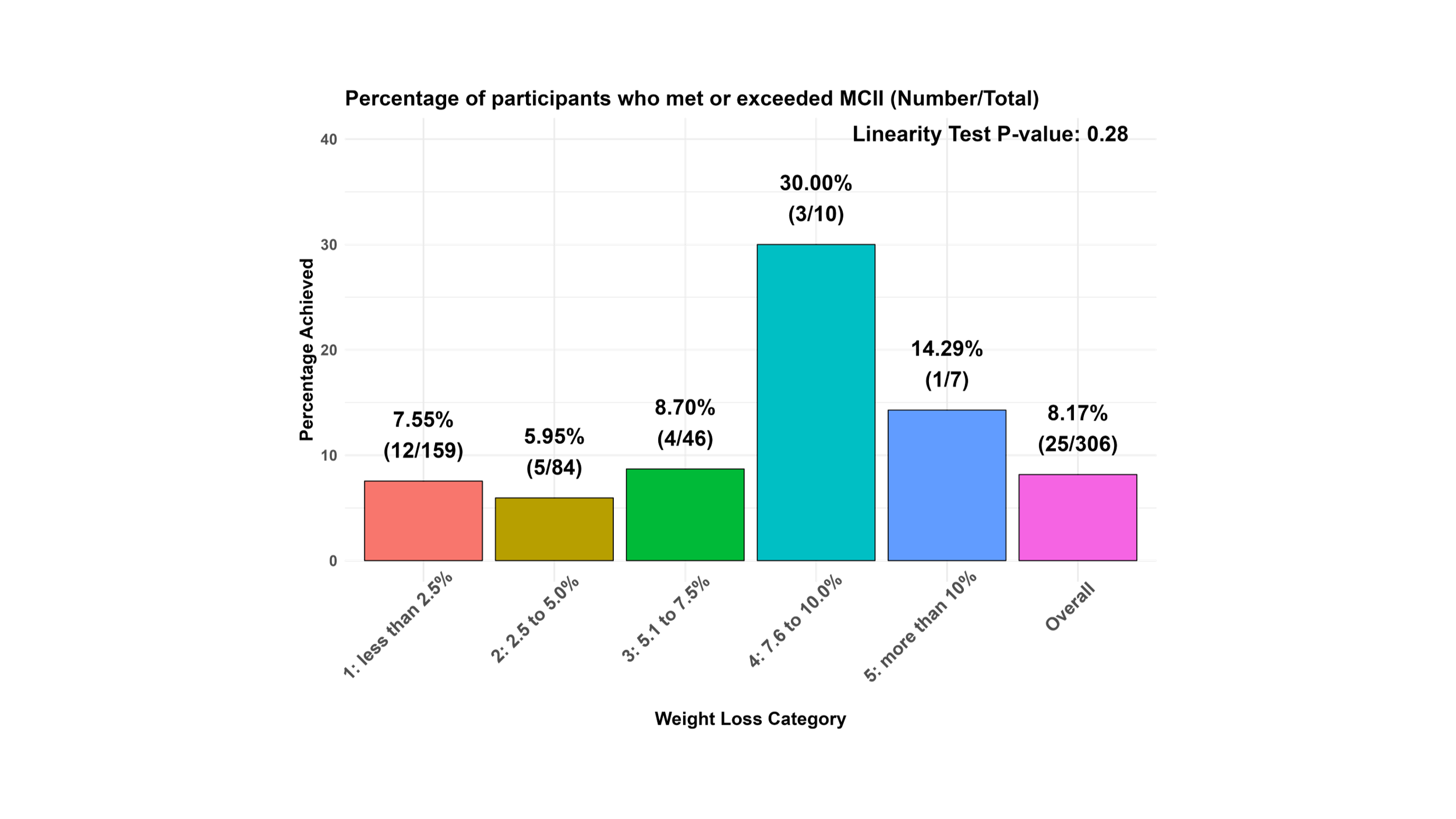


Figure S5. Sensitivity analysis. Dose-response relationship between the categories of percentage body weight change and changes in HOOS scores using multiple imputations for missing values. Linearity tests were adjusted for sex and baseline values of age, weight, and respective HOOS subscale scores. P-values less than 0.05 signify a linear dose-response relationship. A positive difference for change in Hip Disability and Osteoarthritis Outcome Score (HOOS) subscales indicates an improvement. SD: Standard Deviation.


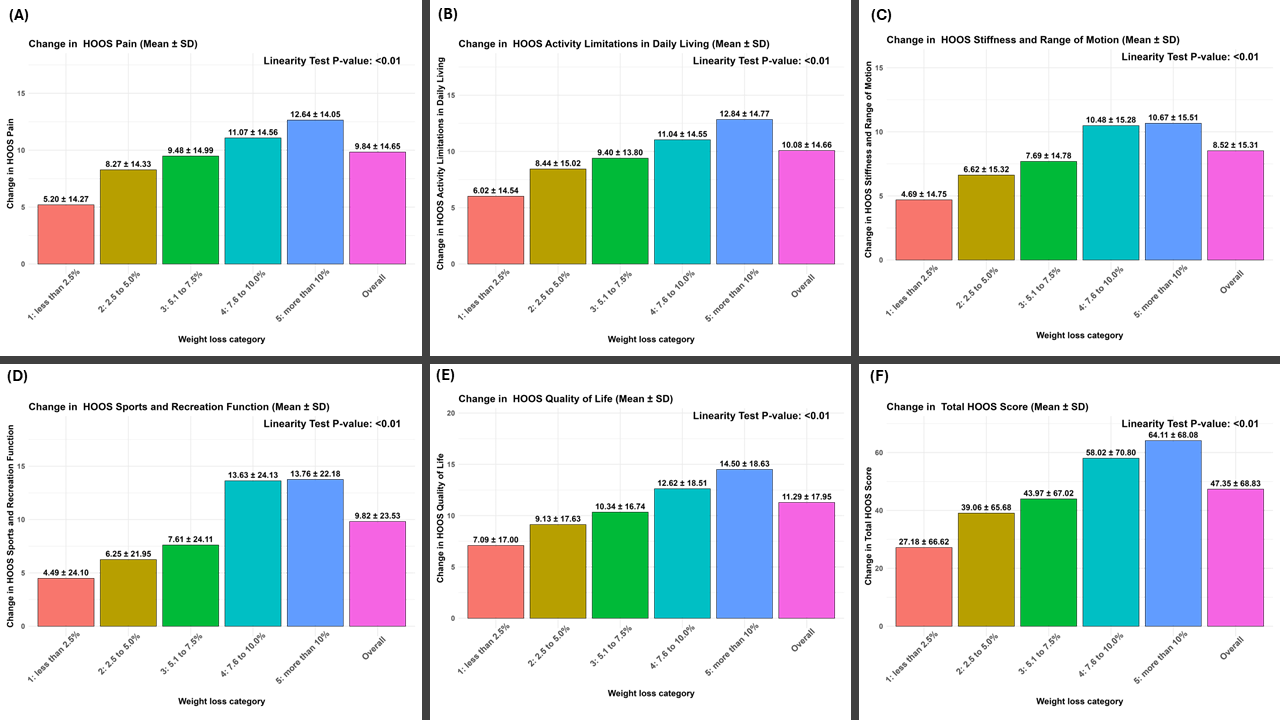


Figure S6. Sensitivity analysis. Dose-response relationship between the categories of percentage body weight change and the percentage of participants meeting or exceeding the MCII threshold for WOMAC function using multiple imputations for missing values. The linearity test was not adjusted. A P-value less than 0.05 signifies a linear dose-response relationship. MCII: Minimal clinically important improvement; WOMAC: Western Ontario and McMaster Universities Osteoarthritis Index.


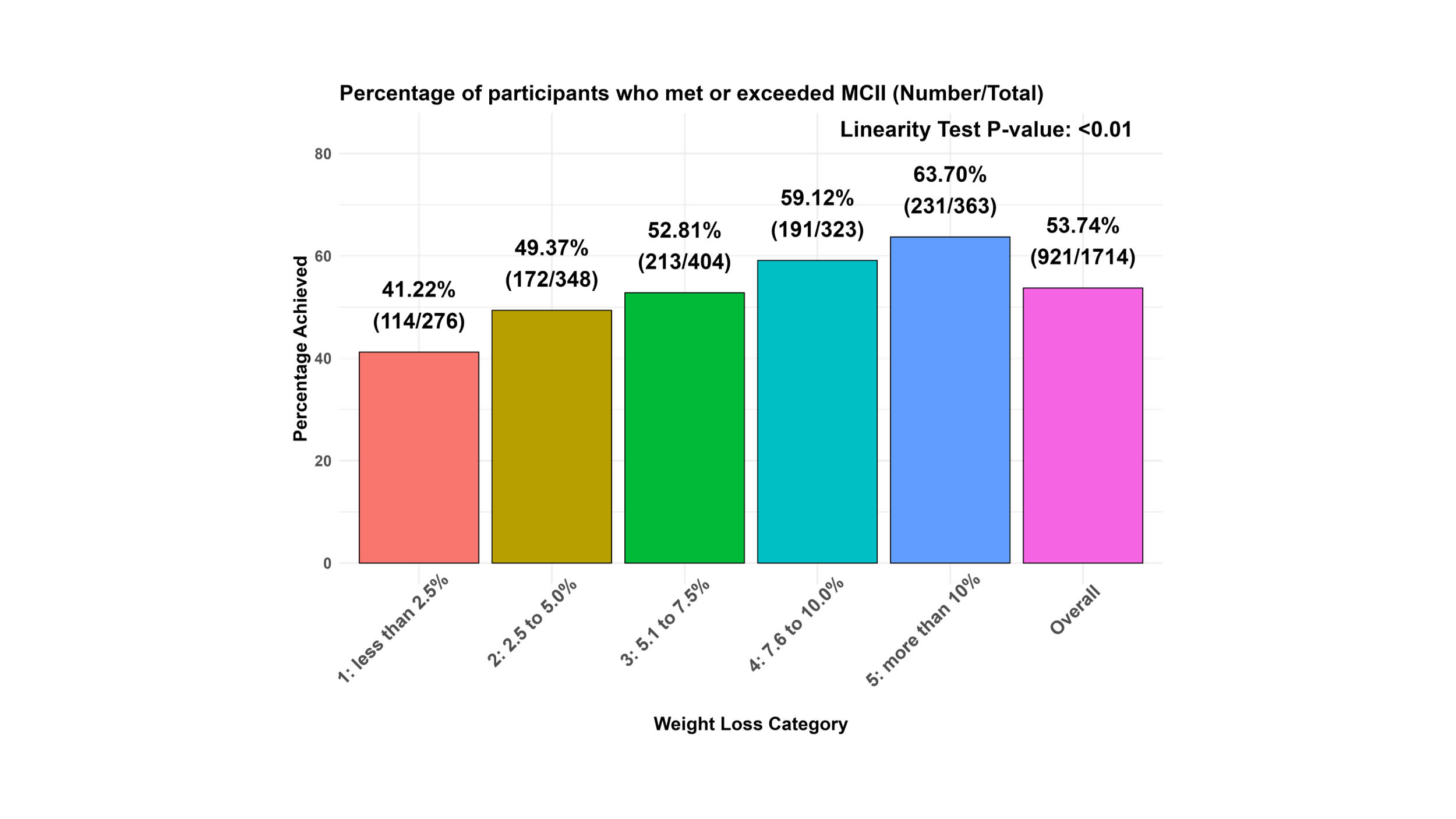


Table S1. Weight loss during the 18-week weight loss phase. OAHWFL: Osteoarthritis Healthy Weight for Life Weight Loss Program, SD: Standard Deviation.

| **Weight loss category** | **Participants who completed the 18-week weight loss phase of the OAHWFL program** | | **Participants who did not complete the 18-week weight loss phase of the OAHWFL program** | |
| --- | --- | --- | --- | --- |
|  | **Total (%)** | **Weight loss kg (%) mean ± SD** | **Total** | **Weight loss kg (%) mean ± SD** |
| All | 1,408 (100) | 7.19 ± 4.26  (7.55 ± 3.99) | 306 (100) | 2.63 ± 3.78  2.67 ± 3.69 |
| ≤2.5% | 117 (8.3) | 0.73 ± 1.55  (0.78 ± 1.56) | 159 (52.0%) | 0.44 ± 3.41  0.46 ± 3.39 |
| >2.5-5.0% | 264 (18.8) | 3.65 ± 0.99  (3.89 ± 0.77) | 84 (27.4%) | 3.51 ± 1.09  3.6 ± 0.77 |
| >5.0-7.5% | 358 (25.4) | 5.83 ± 1.24  (6.25 ± 0.73) | 46 (15.0%) | 5.91 ± 1.36  6.01 ± 0.65 |
| >7.5-10.0% | 313 (22.2) | 8.23 ± 1.53  (8.70 ± 0.71) | 10 (3.3%) | 8.44 ± 1.56  8.34 ± 0.66 |
| >10% | 356 (25.3) | 12.39 ± 3.72  (12.76 ± 2.42) | 7 (2.3%) | 11.91 ± 2.97  11.69 ± 0.93 |

Table S2. Sensitivity analysis. Relationship between percentage body weight change as a continuous variable and changes in HOOS scores using multiple imputations for missing values. * Adjusted for sex, and the baseline measures of age, weight, and respective Hip Disability and Osteoarthritis Outcome Score (HOOS) scores. The β coefficients were calculated and reported based on 1% weight loss from baseline at the 18-week weight loss phase. CI: Confidence Intervals.

| **Outcome** | **Weight loss (%)**  **β (95% CI)** | **P-value*** |
| --- | --- | --- |
| Change in HOOS Pain subscale after weight loss  [% change from baseline] | 0.57 (0.41 to 0.74)  [1.06 (0.69 to 1.43)] | **< 0.001**  **[< 0.001]** |
| Change in HOOS Activity Limitations in Daily Living subscale after weight loss  [% change from baseline] | 0.55 (0.38 to 0.73)  [0.96 (0.55 to 1.38)] | **< 0.001**  **[< 0.001]** |
| Change in HOOS Stiffness and Range of Motion subscale after weight loss  [% change from baseline] | 0.48 (0.30 to 0.65)  [1.13 (0.73 to 1.54)] | **< 0.001**  **[< 0.001]** |
| Change in HOOS Sports and Recreation Function subscale after weight loss  [% change from baseline] | 0.82 (0.55 to 1.09)  [2.54 (1.15 to 3.92)] | **< 0.001**  **[< 0.001]** |
| Change in HOOS Hip-related Quality of Life subscale after weight loss  [% change from baseline] | 0.65 (0.44 to 0.86)  [1.93 (0.82 to 3.04)] | **< 0.001**  **[< 0.001]** |
| Change in Total HOOS score after weight loss  [% change from baseline] | 3.15 (2.36 to 3.94)  [1.37 (1.00 to 1.74)] | **< 0.001**  **[< 0.001]** |
